# Primary Impacts of the Fungal Toxin Sporidesmin on HepG2 Cells: Altered Cell Adhesion without Oxidative Stress or Cell Death

**DOI:** 10.3390/toxins13030179

**Published:** 2021-02-28

**Authors:** Magalie Boucher, T. William Jordan

**Affiliations:** 1 Centre for Biodiscovery and School of Biological Sciences, Victoria University of Wellington, Wellington PO Box 600, New Zealand; Magalie.Boucher@pfizer.com; 2 Drug Safety Research and Development, Pfizer Inc., Cambridge, MA 02139, USA

**Keywords:** cell adhesion, hepatobiliary injury, HepG2 cells, oxidative stress, sporidesmin, two-dimensional electrophoresis

## Abstract

The fungal metabolite sporidesmin is responsible for severe necrotizing inflammation of biliary tract and liver of livestock grazing on pasture containing spores of *Pithomyces chartarum* that synthesizes the toxin. The toxin is secreted into bile causing the erosion of the biliary epithelium accompanied by inflammation and damage to surrounding tissues. Toxicity has been suggested to be due to cycles of reduction and oxidation of sporidesmin leading to oxidative damage from the formation of reactive oxygen species. The current work is the first test of the oxidative stress hypothesis using cultured cells. Oxidative stress could not be detected in HepG2 cells incubated with sporidesmin using a dichlorodihydrofluorescein diacetate assay or by use of two-dimensional electrophoresis to search for oxidized peroxiredoxins. There was also no evidence for necrosis or apoptosis, although there was a loss of cell adhesion that was accompanied by the disruption of intracellular actin microfilaments that have known roles in cell adhesion. The results are consistent with a model in which altered contact between cells in situ leads to altered permeability and subsequent inflammation and necrosis, potentially from the leakage of toxic bile into surrounding tissues. There is now a need for the further characterization of the damage processes in vivo, including the investigation of altered permeability and mechanisms of cell death in the biliary tract and other affected organs.

## 1. Introduction

Sporidesmin is a potent cytotoxin synthesized by the saprophytic fungus *Pithomyces chartarum*. The ingestion of *P. chartarum* spores affects the health and production of livestock grazing on contaminated pasture. Although toxigenic strains of the fungus have a wide international distribution, the known impacts are greatest in New Zealand because of the predominance of pasture-based agriculture [1]. Toxin-mediated hepatobiliary injury results in photosensitization that is secondary to impaired excretion of the chlorophyll metabolite phytoporphyrin. The resulting disease was named facial eczema reflecting damage to the face and ears due to phototoxic reactions of phytoporphyrin in skin exposed to sunlight.

Damage to the biliary tract and liver is a hallmark of the disease but other organs are also affected [2,3]. An initial phase of patchy hepatocellular necrosis precedes necrotizing inflammation in the biliary tract and liver, followed by chronic biliary obstruction and consequential hepatocellular necrosis that is marked within four days of exposure. Medium to large size bile ducts are most affected, like human sclerosing cholangitis. Mortimer (1963) observed the leakage of bile into spaces around the bile ducts in sheep experimentally dosed with sporidesmin and postulated that altered permeability of the biliary tract was implicated in the necroinflammatory changes [2].

**Figure 1:**
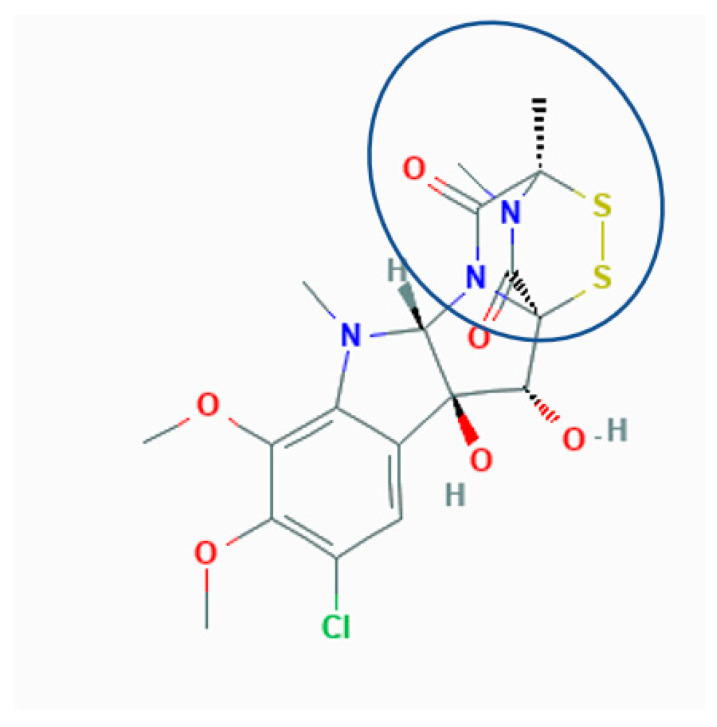
Structure of sporidesmin (sporidesmin A). The reactivity of the sulfur bridge is associated with the molecular environment of the dioxopiperazine nucleus (circled).

The molecular basis of tissue damage and cell death caused by sporidesmin is not clearly established. This toxin is a member of the epidithiodioxopiperazine family of fungal metabolites [4] that contain a chemically reactive [5] sulfur-bridged dioxopiperazine ring that is the toxic moiety (Figure 1).

Toxicity is due to the reactivity of the sulfurs that have been suggested to either covalently modify protein cysteine residues to form protein-toxin mixed disulfides [6], or to generate reactive oxygen species (ROS) during oxidative recycling between the disulfide and dithiol forms of the toxins [7]. The formation of mixed disulfides has been detected following the incubation of sporidesmin, or other epidithiodioxopiperazines including synthetic model compounds, with purified proteins where there is specificity for what proteins and which cysteine residues are modified [8]. Postulated mechanisms of action therefore include a specific reaction with some proteins, or more widespread injury through the reaction of ROS potentially with DNA, proteins and lipids. Neither mechanism has been convincingly established using cultured cells or in vivo.

Sporidesmin is cytopathic to a range of cultured cells [9]. Effects include a loss of attachment of cells accompanied by the disruption of the cytoplasmic actin microfilament network [10]. Among the few other studies of the effects of sporidesmin on cultured cells, the apoptosis of peritoneal macrophages has been reported [11]. In comparison, there are numerous reports of oxidative stress and apoptosis or necrosis in mammalian cells incubated with the structurally-related compound gliotoxin that also contains the toxic sulfur-bridged dioxopiperazine ring and is synthesized by *Aspergillus fumigatus* and other fungi [12].

Although it is possible to assume that cell death in vivo results from oxidative stress or other direct toxicity of sporidesmin, liver cells cultured with the toxin do not appear to undergo cell death [10]. Furthermore, the concepts of cell death have undergone substantial revision and expansion over the past decade. Necrosis, once thought to be an unregulated response to toxic insult, is now seen to include several forms regulated by multiple pathways [13]. Similarly, apoptosis resulting in the removal of cells through scavenging or secondary necrosis has been expanded to include anoikis in which the molecular and cellular events typical of apoptosis result from the disruption of cellular interactions with the extracellular matrix. These shifts in understanding are relevant to cell injury and death caused by sporidesmin. We have therefore used the liver HepG2 cell line as a model to test for oxidative stress and cell death. Experimental conditions included the incubation of cells with 1 μg/mL sporidesmin which is the probable maximum concentration in bile [14].

## 2. Results

### 2.1. Microscopy

**Figure 2:**
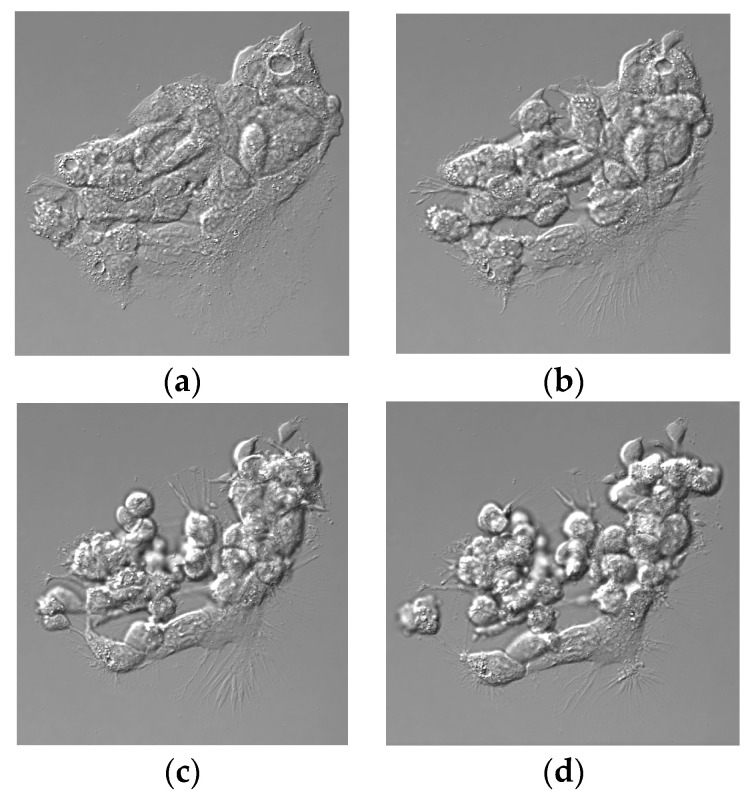
Differential interference contrast (DIC) images of HepG2 cells incubated with 1 µg/mL sporidesmin for 3 h. Cells at time 0 (**a**), 1 (**b**), 2 (**c**), and 3 (**d**) h. Time lapse movies are in the Supplementary Data. There was the rounding up and shrinkage of individual cells prior to the detachment of individual cells and sheets of cells.

Experiments were carried out using monolayer cultures of HepG2 cells at approximately 80% confluence containing multiple colonies of cells. The time-lapse microscopy of control cultures without added sporidesmin showed motion that apparently affected the shapes of individual cells without change in the relative position within the cell mass and without evidence of detachment from the culture plastic (Supplementary Video). In cultures exposed to sporidesmin, there was a progressive loss of contact between cells, shrinkage and rounding up of individual cells, and a release of sheets of cells and detached individual cells into the culture medium within 3 h (Figure 2, and Supplementary Video). Filopodia that anchored cells to the tissue culture substrate became prominent during detachment. A loss of contact occurred within some regions of the cell mass while cell–cell contact was apparently retained in others. Some cells appeared to detach from their neighbors and became free floating prior to the detachment of islands of cells from the tissue culture plastic. Incubation of HepG2 cells with 1 or 4 µg/mL sporidesmin for up to 6 h did not affect cell viability (1 µg/mL 150 ± 16, 4 µg/mL 141 ± 7: % of control, mean ± SD, *n* = 3) measured using an MTT (3-[4,5-dimethylthiazole-2-yl]-2,5-diphenyltetrazolium bromide) assay.

**Figure 3:**
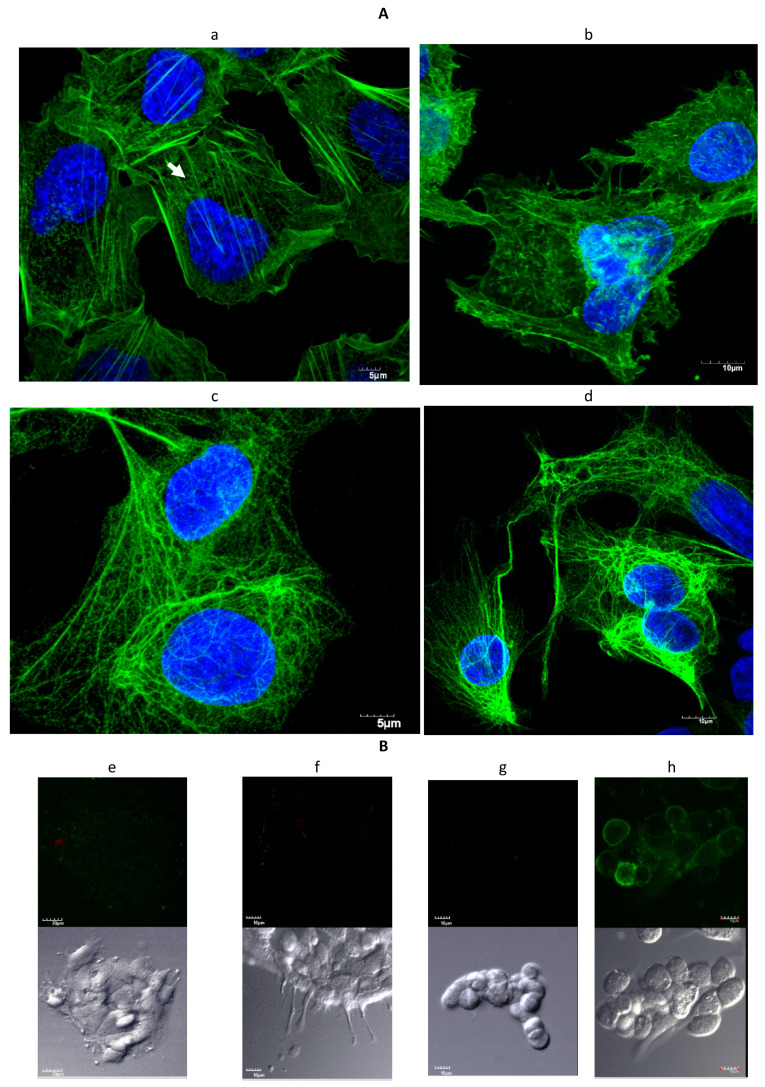
(**A**) Confocal microscopy of actin (**a**,**b**) or cytokeratin (**c**,**d**) filaments in HepG2 cells. Control cells (**a**,**c**) incubated without sporidesmin contained cytoplasmic filaments of actin (arrow) and a network of cytokeratin filaments. Cells incubated with 1 µg/mL sporidesmin for 2 h showed (**b**) a loss of cytoplasmic actin filaments without the collapse of the cytokeratin network (**d**). (**B**) Cells stained with Annexin-V-FLUOS upper panels to detect apoptosis or necrosis. Lower panels are the corresponding DIC images. Positive staining for apoptosis or necrosis was not detected in the control cells (**e**) or in adherent (**f**) or detached (**g**) cells after exposure to 1 µg/mL sporidesmin for 3 h. There was positive control staining of apoptotic cells (**h**) in cultures that had been incubated with yessotoxin.

Immunofluorescent staining of control HepG2 cultures showed cytoplasmic actin microfilaments and networks of cytokeratin intermediate filaments (Figure 3A). A loss of cytoplasmic actin microfilaments occurred in cultures incubated with sporidesmin and there was redistribution of actin to the cortical region underlaying the plasma membrane. The staining of cytoplasmic actin filaments was less obvious in HepG2 cells compared to the previously reported primary cultures of biliary tract cells [15] but there was a similar pattern of loss of cytoplasmic filaments in HepG2 cells and biliary tract cells cultured with sporidesmin. Cytokeratin filaments did not appear to be affected in cells incubated with the toxin apart from an extension of the network into retracting regions of cells.

### 2.2. Staining with Annexin-V-FLUOS

Further evidence for possible deleterious effects of sporidesmin was examined by staining cell cultures with Annexin-V-FLUOS that binds to phosphatidyl serine exposed at the surface of cells during the early stages of apoptosis. Inclusion of propidium iodide in the reagent detects necrosis after this fluorescent dye crosses damaged cellular membranes and stains nuclear DNA. Method development included the 15 min exposure of HepG2 cultures to UV that resulted in extensive staining for apoptosis and necrosis (not shown). Cell death, either apoptosis or necrosis, was not detected in adherent or detached cells from HepG2 cultures incubated with 1 µg/mL sporidesmin for 3 h (Figure 3B). Positive control staining at the plasma membrane of apoptotic cells was obtained using HepG2 cells incubated with 1.4 µM yessotoxin as previously described [16].

### 2.3. Oxidative Stress

**Figure 4:**
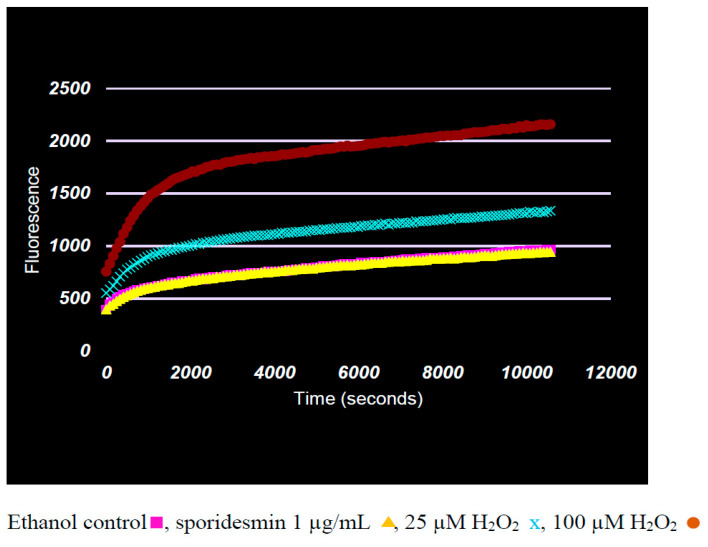
Dichlorodihydrofluorescein diacetate (DCFDA) assay for the detection of reactive oxygen species (ROS) in cells incubated with 1 µg/mL sporidesmin or 25 or 100 µM hydrogen peroxide. Enhanced fluorescence from the oxidation of dichlorofluorescein was detected in cultures containing hydrogen peroxide but did not increase above basal levels in cultures containing sporidesmin. Results are the means and SDs of triplicates. Results for controls without ethanol (not shown) overlaid the ethanol controls.

Evidence for oxidative stress was examined using the dichlorodihydrofluorescein diacetate (DCFDA) assay that detects a range of oxidative species [17] including superoxide, hydrogen peroxide and hydroxyl radical that have been proposed to result from the oxidative recycling of the disulfide bridge of sporidesmin [7,18]. There was basal increase in fluorescence in HepG2 cells cultured for 3 h in the absence or presence of 0.1% ethanol, but that was not enhanced in cultures containing 1 µg/mL sporidesmin (Figure 4). In contrast, positive controls containing 25 or 100 µM hydrogen peroxide showed the expected time-dependent increase in fluorescence. These results indicated that oxidative stress was not pronounced during the 3 h incubation with sporidesmin in which a loss of adhesion and the disruption of cytoskeletal actin microfilaments occurred.

**Figure 5:**
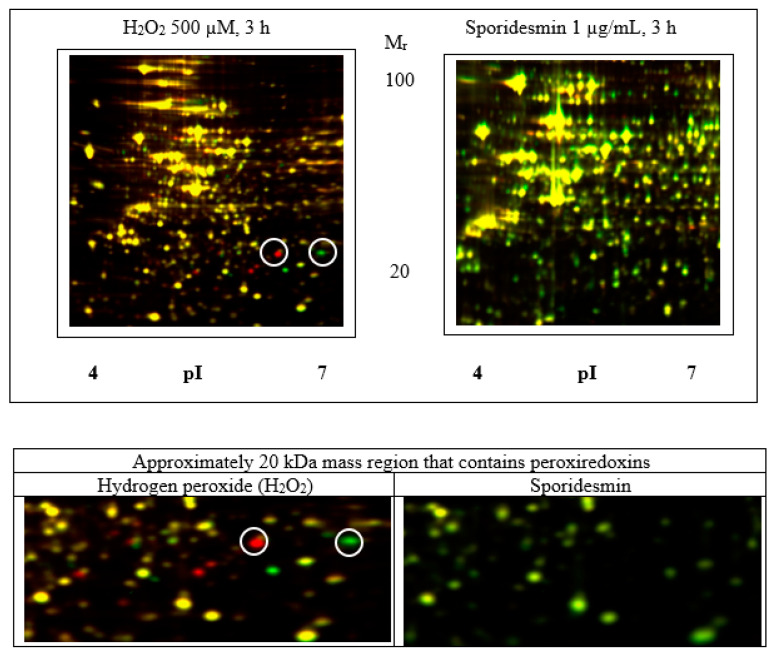
The two-dimensional electrophoresis of proteins from HepG2 cells that had been incubated with 1 µg/mL sporidesmin or 500 µM hydrogen peroxide for 3 h. The differential in-gel electrophoresis (DIGE) labelling design facilitates the detection of changes in protein abundance compared to control incubates with or without 0.1% ethanol. Extracts of cells incubated with hydrogen peroxide showed multiple changes including a decrease in the abundance of native peroxiredoxins with a gain of acidic isoforms that are expected to be the products of cysteine oxidation. Lower panels show the approximately 20 kDa mass regions containing peroxiredoxins. Circled peroxiredoxin 6 isoforms correspond to the oxidized (left) and native (right) proteins that, respectively, increased or decreased after culture with hydrogen peroxide. There were no detected changes in the abundance of any protein spots from cells that had been incubated with sporidesmin.

Further investigation of the potential effects of oxidative stress was carried out using two-dimensional electrophoresis of cell extracts to search for evidence of the oxidation of protein cysteine thiols including the sulfonic (SO_3_H) acid derivatives of peroxiredoxins [19]. The experimental design used differential in-gel electrophoresis (DIGE) minimal labelling with CyDyes to compare treated and control samples on gels containing four biological replicates for each condition. Isoelectric focusing from pI 4–7 and 6–11 was used as the first dimension (horizontal) separation and was followed by SDS polyacrylamide gel electrophoresis for size-based separation in the second (vertical) dimension. The oxidation of cysteine thiols is expected to result in acidic isoforms in the focusing dimension. Cells incubated with 500 μM hydrogen peroxide for 3 h were used as a positive control that showed acidic pI shifts of some proteins indicative of oxidative stress (Figure 5). Supplementary Figures S1 and S2 show that peroxiredoxin 1 (Supplementary Figure S3) and 6 (Supplementary Figure S4) isoforms were present as pairs of spots with decrease in the more basic and increase in the more acidic forms in peroxide-treated cells. The relative positions of peroxiredoxins on the gels corresponded with the predicted pI and size of the native proteins, for example peroxiredoxin 1 pI 8.3/22 kDa, peroxiredoxin 6 pI 6/25 kDa (Supplementary Table S1). In contrast, the exposure of cells to 1 μg/mL sporidesmin did not result in the observable oxidation of peroxiredoxins or any other significant changes in protein position or abundance.

## 3. Discussion

The goal of this work was to gain further insight into the cellular effects of sporidesmin including testing for the formation of ROS and for evidence of necrosis or apoptosis within the time frame of other cellular change, namely detachment of cultured cells. We suggest that the observed loss of contact between cells and of cells to the culture plastic may be similar to the changes in vivo that affect the permeability of the biliary tract and other tissues. It is, however, likely that there are multiple toxic targets of sporidesmin affecting a wide range of physiological functions due to the promiscuous reactivity of the toxin sulfurs [6]. Therefore, although this discussion includes the impact on cell adhesion that modulates the connection of cells to each other and to the extracellular matrix, we do not preclude other effects of sporidesmin.

### 3.1. Oxidative Stress

Oxidative stress has been assumed to be a major contributor to sporidesmin-induced tissue pathology since Munday’s work on the formation of ROS ([7], and subsequent publications) using aqueous incubates. Although others have reported the detection of ROS in cells cultured with gliotoxin, there are no positive or negative reports of oxidative stress caused by sporidesmin using cell cultures or in vivo. We therefore used two assays to test for evidence of oxidative stress in HepG2 cells. Both were negative with sporidesmin although positive for cultures incubated with hydrogen peroxide. In the DCFDA assay, oxidation was greatest in the first 30 min in cells incubated with hydrogen peroxide, and then increased parallel to basal levels. Others have demonstrated the decomposition of peroxide within 15 min in cell cultures [20], which may account for the decrease in oxidation at later times. Sporidesmin did not enhance the oxidation of dichlorofluorescein compared to the basal increase in fluorescence in controls without sporidesmin or hydrogen peroxide.

Three-hour incubations with sporidesmin or hydrogen peroxide were used for cultures examined by two-dimensional electrophoresis to correspond to the time of sporidesmin-induced cellular changes detected by differential interference contrast (DIC) microscopy. Shorter incubations with hydrogen peroxide were not examined although based on the DCFDA results, it was possible that the substantial oxidation of proteins occurred within the first hour. Rabilloud et al. established that the oxidation of active site cysteines of peroxiredoxins is a sensitive measure of oxidative stress that could be detected by acidic shifts of the oxidized proteins on two-dimensional electrophoresis gels [19]. In that study, the persistence of the oxidized forms varied between various peroxiredoxins but was clearly detectable several hours after the transfer of cells to fresh culture medium without the stress agent. In the current study, oxidized proteins including peroxiredoxins 1 and 6 were detected in cells cultured with 500 µM hydrogen peroxide while there were not any significant changes in protein abundance caused by sporidesmin. Oxidized proteins including peroxiredoxins 1 and 6 have previously been detected by two-dimensional electrophoresis of HepG2 cells cultured with 500 µM hydrogen peroxide [21]. The absence of protein change in HepG2 cells exposed to sporidesmin was unexpected as many drugs and toxicants have characteristic proteomic signatures that reflect mechanisms and tissue responses [22]. It is possible that 3 h exposure was not sufficient to perturb individual proteins and networks of proteins, but we did not detect for example protein changes that have previously been associated with apoptosis caused by other compounds [16,23]. Future work could include the investigation of the possible oxidation of protein cysteines using DIGE, or gel-free mass spectrometry of protein digests [20], of tissue from animals exposed to sporidesmin.

There are links between oxidative stress and apoptosis in various systems [24], but Halliwell has cautioned that oxidative stress mechanisms and their stimulatory effects on apoptosis may be overrepresented in cell cultures in part due to the high concentration of oxygen and prooxidants including transition metal ions, and low antioxidants compared to tissue [25]. Notwithstanding, a key feature of the current work is that cellular change occurred in the absence of detectable oxidative stress.

### 3.2. Cytotoxicity and Cell Death

A loss of adhesion of adherent cell cultures is characteristic for both sporidesmin and gliotoxin [10]. The current work demonstrated that a loss of cell adhesion and disruption of actin microfilaments in HepG2 cells occurred in the apparent absence of apoptosis or necrosis or of generation of ROS within the 3 h period in which there was a perturbation of attachment among cells and of cells to the culture substrate.

For comparison, there are numerous reports of oxidative stress and cell death in cells exposed to gliotoxin [5,12,26] but only a single report of morphological change and fragmentation of DNA characteristic of apoptosis in cells cultured with sporidesmin [11]. There may be some differences between the cellular effects of sporidesmin and gliotoxin, although reactions of the sulfur bridge are central for both toxins. For example, gliotoxin rapidly induced the detachment of single cells from adherent cell lines and was associated with anoikis [27] but the detachment of sheets of cells was more common using cultured biliary tract cells [15] exposed to sporidesmin and in the current study. Differences among cell types in sensitivity and response to the toxins, and differences in culture conditions including toxin concentration and the duration of exposure, may also be relevant to the comparison of the effects of sporidesmin and gliotoxin and to the assessment of pathology in vivo caused by these toxins.

### 3.3. Cell Adhesion

The disruption of the actin cytoskeleton may be linked to altered cell adhesion, either as a cause or consequence of altered cell contact, through networks including proteins that span the plasma membrane and interact with the extracellular matrix and with intracellular adhesion complexes that bind and regulate actin [28]. Effects on cell adhesion and disruption of the actin cytoskeleton have been related to sensitivity to liver damage in cells cultured from the biliary tract of sheep selected for resistance to sporidesmin [15] and to the disruption of pulmonary epithelium by gliotoxin during invasive aspergillosis [29]. Suggested mechanisms of gliotoxin-mediated disruption of actin microfilaments have been related to covalent adduction of cell surface cysteines of integrin proteins that span the plasma membrane [27] or to the modulation of the cytoplasmic actin-regulating protein cofilin [30].

This knowledge is potentially relevant to Mortimer’s conclusion that the disruption of the epithelial lining of the biliary tract is characteristic of sporidesmin-induced injury and precedes necrotizing inflammation of surrounding tissue [2]. This view is also consistent with the time course of liver and biliary tract injury in mice dosed with sporidesmin where the earliest effects were subtle changes in the morphology of the epithelium of the gallbladder wall and extrahepatic ducts at two days, prior to the extensive necrosis of the intra- and extra-hepatic ducts, gallbladder wall and liver at four days [31].

Current general concepts of biliary pathology include differences in the sensitivity to the damage of the cells (cholangiocytes) lining the intra- and extra-hepatic bile ducts due to differences in gene expression and molecular composition, the altered permeability of biliary epithelium resulting in attack on surrounding tissues by bile acids and other irritant molecules leaking from bile, and immune responses including the secretion of cytokines by activated cholangiocytes [32]. The altered adhesiveness of cells exposed to sporidesmin in culture may therefore be representative of changes that affect the integrity of cells exposed to sporidesmin circulating in the bile and blood of livestock and may be generally relevant to human diseases of the liver and gut that are characterized by altered permeability and inflammation [33].

**Figure 6:**
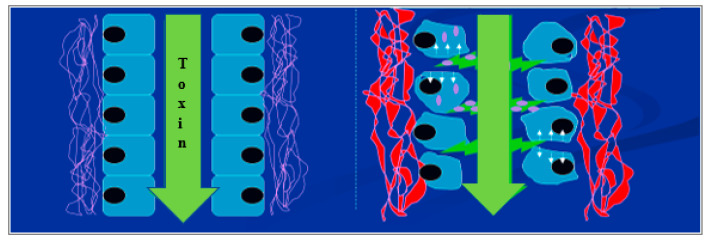
Proposed model for changes in cell contact and adhesion. Sporidesmin in the biliary tract or circulation alters contact between cells leading to enhanced permeability and subsequent tissue damage.

In the proposed model (Figure 6), sporidesmin disrupts contacts between cells resulting in enhanced permeability. Although the key protein targets of sporidesmin have not been identified, the remodelling of the actin cytoskeleton potentially impacts the integrity of junctional complexes between cells [34]. The disruption of junctional complexes has been associated with hepatobiliary pathologies including primary sclerosing cholangitis [35] which like sporidesmin affects medium and large size bile. The molecular events by which sporidesmin may alter cell contact remain to be elucidated. It is also unknown whether the subsequent extensive necrosis of epithelium and surrounding tissues are direct toxicological actions of sporidesmin, although it seems likely that they are secondary to the disruption of the epithelia, with toxic effects of bile leaking into surrounding tissue.

## 4. Conclusions

Our results suggest that primary tissue injury may not be due to the generation of ROS by the oxidative recycling of sporidesmin or to interactions of the toxin that directly activate cell death processes. There is now a need for the further characterization of the damage processes in vivo including the investigation of the altered permeability in the biliary tract and other affected organs. This could include tests for oxidative stress including oxidized peroxiredoxins, the molecular analysis of cell death pathways, and the identification of the molecular targets and interactions of sporidesmin. In addition to the generation of fundamental knowledge about disease processes there may be implications for the design of control strategies that protect livestock from the deleterious effects of the ingestion of fungal spores containing sporidesmin.

## 5. Materials and Methods

### 5.1. Cell Culture

Human HepG2 hepatoblastoma cells (HB-8065) from the American Type Culture Collection (Manassas, VA, USA) were cultured at 37 °C and 5% CO_2_ in air, in RPMI 1640 (GIBCO, Invitrogen Carlsbad, CA, USA) supplemented with 10% fetal calf serum (ICPbio Ltd., Auckland, New Zealand) and 1% penicillin–streptomycin (5000 units/mL penicillin G sodium and 5000 µg/mL streptomycin sulphate in 0.85% saline) (GIBCO, Invitrogen Carlsbad, CA, USA).

Sporidesmin (Mr 473, aqueous solubility 30 ppm, >98% purity, from AgResearch, Ruakura, Hamilton, New Zealand) was dissolved in absolute ethanol and added to cell cultures to give 1 µg/mL concentrations of the toxin at 0.1% ethanol. Controls without sporidesmin included the presence or absence of 0.1% ethanol.

### 5.2. Microscopy

Microscopy methods were as described by Lindsay et al. [15]. The time-lapse microscopy of HepG2 cells cultured in the presence or absence of sporidesmin was carried out at 5 min intervals for 3 h using an AX70 photomicroscope (Olympus, Tokyo, Japan) with a DP70 camera (Olympus) and DP Controller 1.2.1.108 software. A FV1000 Confocal Laser Scanning Microscope was used for the immunofluorescent detection of actin microfilaments and cytokeratin intermediate filaments in formalin-fixed Triton X-100 permeabilized cells. Actin microfilaments were stained with Alexa Fluor 488 phalloidin (Molecular Probes, Eugene, OR, USA). Intermediate filaments were stained by reaction with anti-cytokeratin clone AE1/AE3 (DAKO, Glostrup, Denmark) and detected using secondary antibody (Alexa Fluor 488 goat anti-mouse IgG (H+L); Molecular Probes). Cell nuclei were stained with 4′,6-diamidino-2-phenylindole (DAPI).

### 5.3. Apoptosis

Apoptosis was examined using Annexin-V staining of phosphatidylserine at the surface of apoptotic cells as previously described [16]. HepG2 cells cultured in the presence or absence of sporidesmin, or of 1.4 µM yessotoxin as a positive control, were stained for 15 min with Annexin-V-FLUOS (Roche, Penzburg, Germany) in 140 mM NaCl, 5 mM CaCl_2_, 10 mM HEPES–NaOH pH 7.4 containing propidium iodide. After incubation, cells were washed in the same buffer and images were acquired using confocal microscopy.

### 5.4. Oxidative Stress and Cytotoxicity

HepG2 cells were cultured overnight in 0.1 mL RPMI 1640 with 10% fetal calf serum at 37 °C in 96-well plates (Greiner Bio-one) (5 × 10^4^ cells per well). The next day the medium was removed and after rinsing with phosphate-buffered saline, the cells were incubated in a microplate reader (BMG FLUOstar, BMG LABTECH, Ortenberg, Germany) with 0.1 mL Hanks’ Balanced Salts (Sigma H1387) per well containing 10 µM DCFDA (Merck KGaA, Darmstadt, Germany). After loading cells with DCFDA for 20 min, test conditions were initiated with or without sporidesmin, or with hydrogen peroxide as a positive control. The fluorescence intensity of triplicate cultures was measured continuously for 3 h with excitation of 485 nm and emission of 520 nm.

Cytotoxicity was assessed as metabolic capacity to reduce MTT [36] using cells cultured in 0.1 mL RPMI 1640 as above. Replicate (*n* = 3) cultures were incubated with 1 or 4 µg/mL sporidesmin for 6 h prior to addition of 20 µL 5 mg/mL MTT solution to each well and incubation for 2 h at 37 °C. The insoluble dye product was dissolved and absorbance at 570 nm was measured in a VersaMAX multiwell plate spectrophotometer (Molecular Devices, Sunnyvale, CA, USA).

### 5.5. Two-Dimensional Gel Electrophoresis of Proteins

Quantitative analysis of protein change using two-dimensional DIGE of CyDye-labelled proteins, and identification of proteins by MALDI mass spectrometry, was as previously described [23]. Cells at approximately 80% confluence were incubated in 5 mL RPMI 1640 without serum. Experimental groups were added sporidesmin, controls with or without 0.1% ethanol, or with hydrogen peroxide (four replicates per experimental group). For DIGE analysis, samples for comparison were labelled with Cy3 or Cy5 dyes and a pooled control of all samples was labelled with Cy2 as an internal standard. The protocols for minimal labelling proteins with CyDyes, electrophoresis, DIGE analysis and mass spectrometry are described in Supplementary Figure S1.

## Data Availability

All data are contained in the manuscript.

## Supplementary Materials

The following are available online at https://www.mdpi.com/2072-6651/13/3/179/s1, Figure S1: DIGE analysis of cells incubated with sporidesmin or hydrogen peroxide, Figure S2: 20 kDa region of gels showing location of peroxiredoxins, Table S1: Protein spots matched to peroxiredoxins by MALDI mass fingerprinting, Figure S3: Detected peptides (red) PRX-1, Figure S4: Detected peptides (red) PRX-6, Video S1: Time lapse microscopy of HepG2 cells: (a) control; and (b) sporidesmin.
