# Supplementary material for: Primary Impacts of the Fungal Toxin Sporidesmin on HepG2 Cells: Altered Cell Adhesion without Oxidative Stress or Cell Death
Source: Toxins (Basel). 2021 Feb 28;13(3):179. doi: 10.3390/toxins13030179 (PMC7997482; doi:10.3390/toxins13030179)
Supplement: Supplementary file 1 [file toxins-13-00179-s001.zip › toxins-1109601 supplementary final.pdf]

Primary Impacts of the Fungal Toxin Sporidesmin on HepG2 cells: Altered Cell Adhesion Without Oxidative Stress or Cell Death

Magalie Boucher and T. William Jordan

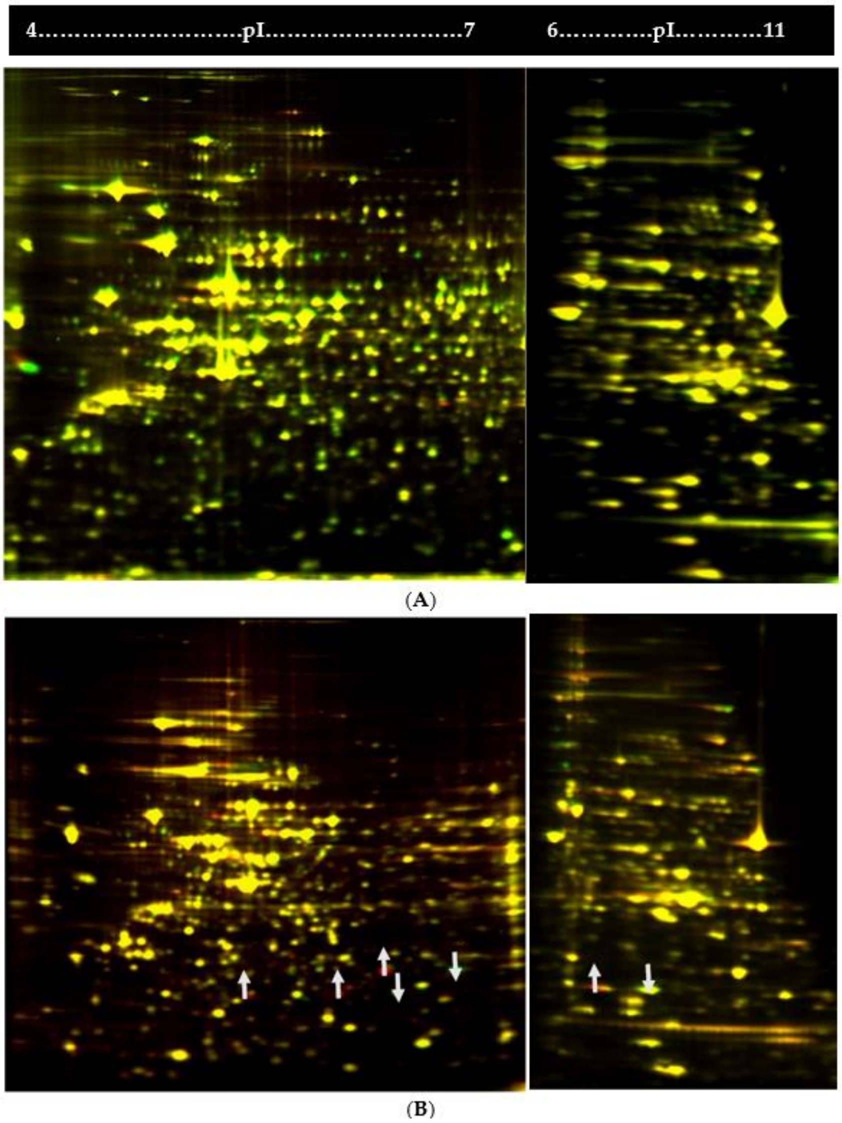

Figure S1. DIGE analysis of cells incubated with sporidesmin or hydrogen peroxide. (**A**) Sporidesmin 1 μg/mL for 3 h; (**B**) Hydrogen peroxide 500 μM for 3 h.

Representative images of CyDye-labelled proteins, showing comparison of A. Cells incubated with sporidesmin compared to controls, B. Cells incubated with H_2O_2 compared to controls. Yellow, protein spots with similar abundance between test and control. Green, spots with decreased abundance (↓) in test (sporidesmin or H_2O_2) compared to control. Red, spots with increased abundance (↑) in test compared to control (Figure S1). Quantitative analysis using DeCyder software indicated that there were not any significant (>1.5 fold, $p<0.05$) changes in spot abundance between sporidesmin and control (with or without ethanol) cells. In contrast, multiple changes in protein abundance were detected for cells that had been cultured with H_2O_2, including apparent shifts towards more acidic isoforms that may reflect oxidation of cysteine residues.

MALDI mass fingerprinting of tryptic digests of protein spots excised from Coomassie stained gels indicated that the varying spots from the approximately 20 kDa mass range (Figure S2, Table S1) were peroxiredoxin (PRX) proteins. Spots corresponding to PRX-1 (Figure S3) and PRX-6 (Figure S4) were identified from control and H_2O_2-exposed cells. Spots corresponding to the presumptive (more acidic) oxidized forms of PRX-1, 2 and 6 were detected from H_2O_2-exposed cells. Other protein spots were too low abundance for confident identification from the MALDI spectra, including a pair of spots that were more acidic and lower mass that PRX-6 and may be isoforms of PRX-3 (predicted pI/kDa 5.8/21.5, compare predicted pI/kDa 6/25 for PRX-6).

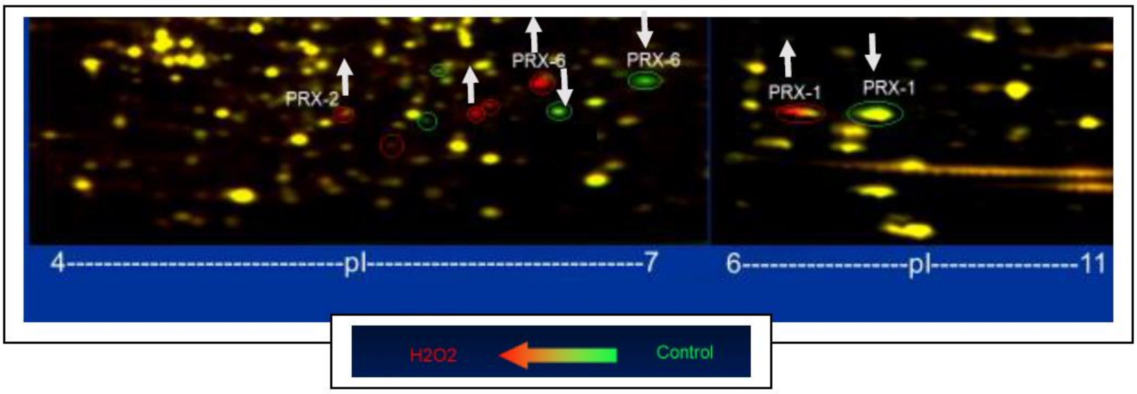

Figure S2. 20 kDa region of gels showing location of peroxiredoxins.

Table S1. Protein spots matched to peroxiredoxins by MALDI mass fingerprinting.

Protein Name	Accession Number	Mascot Score	Coverage %	Calculated pI/kDa
Peroxiredoxin-1	gi|55959887	73	49	8.3/22
Peroxiredoxin-1*		65	42	
Peroxiredoxin-2*	gi|119604714	58	33	5.7/22
Peroxiredoxin-6	gi|4758638	106	57	6/25
Peroxiredoxin-6*		70	33	

Calculated pI and size are based on unmodified protein sequences. Shifts to more acidic pIs are expected for oxidized isoforms. * Predicted oxidized PRXs, matched to sequence databases from the spectra of spots detected from cells incubated with H_2O_2. Tryptic peptides corresponding to the oxidation-sensitive cysteine, for example cys-52 of PRX-1 or cys-47 of PRX-6, were only detected from one protein spot, the basic PRX-6 from cells that had not been incubated with H_2O_2. This peptide was detected at the mass of the expected carbamidomethyl derivative from the reaction of peptide cysteines with iodoacetamide during sample preparation. Mass additions of 48 that would be expected for peptides containing the oxidized cys-SO_3 were not detected for any protein spots from cells incubated with H_2O_2.

Further characterization of the proteins was not carried out as abundance changes were not detected from cells incubated with sporidesmin.

1 MSSGNAK**IGH** **PAPNFK**ATAV MPDGQFKDIS LSDYKGKYVV FFFYPLDFTF
51 VCPTEIIAFS DRAEEFKKLN CQVIGASVDS HFCHLAWVNT PK**KQGGLGPM**
101 **NIPLVSDPKR** **TIAQDYGVLK** **ADEGISFRGL** **FIIDDKGILR** **QITVNDLPVG**
151 **RSVDETLRLV** **QAFQFTDK**HG EVCPAGWKPG SDTIKPDV

Figure S3. Detected peptides (red) PRX-1.

```
  1 MPGGLLLGDV APNFEANTTV GRIRFHDFLG DSWGILFSHP RDFTPVCTTE
 51 LGRAAKLAPE FAKRNVKLIA LSIDSVEDHL AWSKDINAYN CEEPTEKLPF
101 PIIDDRNREL AILLGMLDPA EKDEKGMPVT ARVVFVFGPD KKLKLSILYP
151 ATTGRNFDEI LRVVISLQLT AEKRVATPVD WKDGDSVMVL PTIPEEEAKK
201 LFPKGVFTKE LPSGKKYLRY TPQP
```

Figure S4. Detected peptides (red) PRX-6.

Methods

Protein separation using two-dimensional electrophoresis, the quantitative analysis of protein change using two-dimensional difference gel electrophoresis (2D-DIGE), and the identification of proteins by MALDI mass fingerprinting, were as previously described [1,2].

Cells at approximately 80% confluence were incubated in RPMI without serum. Experimental groups were either added sporidesmin, controls with or without 0.1% ethanol, or $H_2 0_2$ (four biological replicates per experimental group). Following incubation, cells were harvested by scraping, and washed twice by resuspension in SBS and centrifugation at 160 g for 5 min. Cell pellets were vortexed in lysis buffer (30 mM Tris, 7 M urea, 2 M thiourea, 4% CHAPS) to dissolve proteins, and protein concentration was determined using the BioRad Protein Assay (Bio-Rad Laboratories, Hercules, CA, USA. Proteins were precipitated from the extracts using a ProteoExtract® Protein Precipitation Kit (Calbiochem, Merck, KgaA, Darmstadt, Germany), then collected by centrifugation at 10,000× *g* for 10 min.

DIGE Minimal Labelling was carried out according to the manufacturer's instructions (GE Healthcare). Samples for comparison were labelled with Cy3 or Cy5 dyes and a pooled control of all samples was labelled with Cy2 as an internal standard. Prior to labelling the pH of samples in lysis buffer was adjusted to 8.5 with 1.5 M Tris, then 20 µg protein samples were reacted with 80 pmol CyDye for 30 min on ice.

For two-dimensional electrophoresis, the proteins were separated by isoelectric focusing on 7 cm pH 4-7 and 6-11 Immobiline™ DryStrips (GE Healthcare Life Sciences, Uppsala, Sweden) prior to second dimension SDS polyacrylamide gel electrophoresis.

For pH 4–7 separation, proteins dissolved in rehydration buffer (7 M urea, 2 M thiourea, 4% CHAPS, 2% IPG buffer 4–7, 2% DTT) were applied to DryStrips by passive hydration then focused using an IPGphor (GE Healthcare) initially at 300 V for 0.5 h, then 1000 V (gradient) for 0. 5 h and 5000 V for 2 h. For pH 6–11 separation, samples were applied to the strips by cup loading. Focused strips were equilibrated in equilibration buffer (50 mM Tris–HCl, 6 M urea, 30% glycerol, 2% SDS) containing 1% DTT, followed by equilibration buffer containing 2.5% iodoacetamide, for 10 min each, then electrophoresed on 4–12% Bis-Tris NuPAGE gels (Invitrogen, Carlsbad, CA, USA).

Following two-dimensional electrophoresis, the DIGE gels were scanned using a Fujifilm FLA-5100 (Fuji Photo Film, Japan). Cy2 images were acquired using a 473 nm laser and a BPB1/530DF20 emission filter, Cy3 images with a 532 nm laser and a PBG/570DF20 emission filter and Cy5 images with a 635 nm laser and a DBR1/R665 emission filter. Image analysis was carried out with DeCyder™ 2D 6.5 software (GE Healthcare). DeCyder Biological Variation Analysis was used for spot matching and to calculate spot volumes. Statistical analysis was performed using Student's *t*-test (equal variance two-tailed test, $p < 0.05$).

For protein identification, electrophoresed gels were stained with Coomassie Brilliant Blue G-250. Excised protein spots were washed in 50 mM ammonium bicarbonate–methanol (1:1 *v/v*) and digested for 5 h with trypsin (modified sequencing grade; Roche, Mannheim, Germany) in 20 mM ammonium bicarbonate. Peptides extracted into 0.1% TFA–ACN (1:1 *v/v*) were concentrated then resuspended in 2 µL of matrix solution10 mg/mL CHCA in 0.2% TFA–ACN (1:1 *v/v*). MALDI mass fingerprinting was carried out using an Applied Biosystems (Foster City, CA) Voyager DE-Pro mass spectrometer in positive ion mode with monoisotopic masses calibrated against matrix and trypsin peaks. Peptide mass fingerprints were searched against SwissProt 2020_03 Homo sapiens (20,369 sequences) taxonomy using Mascot at http://www.matrixscience.com (accessed on 26 June 2020). Search parameters were the complete modification of cysteine by iodoacetamide and partial modification by methionine oxidation. One missed trypsin cleavage was allowed and peptide tolerance was < 40ppm. Theoretical pI and Mr values for peroxiredoxins were calculated from the peptide sequences using the UniProt *Compute pI/MW* tool.

References

1. Young, C.; Truman, P.; Boucher, M.; Keyzers, R.A.; Northcote, P.; Jordan, T.W. The algal metabolite yessotoxin affects heterogeneous nuclear ribonucleoproteins in HepG2 cells. *Proteomics* **2009**, *9*, 2529-2542.

2. Wilmes, A.; Chan, A.; Rawson, P.; Jordan, T.W.; Miller, J.H. Paclitaxel effects on the proteome of HL-60 promyelocytic leukemic cells: Comparison to peloruside A. *Invest. New Drugs* **2012**, *30*, 121–129.
